# Music Modulates Autonomic Nervous System Activity in Human Fetuses

**DOI:** 10.3389/fmed.2022.857591

**Published:** 2022-04-14

**Authors:** Francesca Massimello, Lucia Billeci, Alessio Canu, Maria Magdalena Montt-Guevara, Gaia Impastato, Maurizio Varanini, Andrea Giannini, Tommaso Simoncini, Paolo Mannella

**Affiliations:** ^1^Department of Clinical and Experimental Medicine, University of Pisa, Pisa, Italy; ^2^Institute of Clinical Physiology, National Research Council of Italy (CNR-IFC), Pisa, Italy

**Keywords:** fetal autonomic nervous system, autonomic nervous system evaluation, fetal nervous system, heart rate variability (HRV), musical stimulation, non-invasive evaluation

## Abstract

**Context:**

Fetal Autonomic Nervous sysTem Evaluation (FANTE) is a non-invasive tool that evaluates the autonomic nervous system activity in a fetus. Autonomic nervous system maturation and development during prenatal life are pivotal for the survival and neuropsychiatric development of the baby.

**Objective:**

Aim of the study is to evaluate the effect of music stimulation on fetal heart rate and specific parameters linked to ANS activity, in particular fetal heart rate variability.

**Methods:**

Thirty-two women between the 32nd and 38th week with a singleton uncomplicated pregnancy were recruited. All FANTE data collections were acquired using a 10-derivation electrocardiograph placed on the maternal abdomen. In each session (5 min basal, 10 min with music stimulus, and 5 min post-stimulus), FANTE was registered. The music stimulus was “Clair de lune” Debussy, played through headphones on the mother’s abdomen (CTR: 31927).

**Results:**

Music does not change the mean value of fetal heart rate. However, indices of total fetal heart rate variability statistically increase (RRsd *p* = 0.037, ANNsd *p* = 0.039, SD2 *p* = 0.019) during music stimulation in comparison to the basal phase. Heart rate variability increase depends mainly on the activation of parasympathetic branches (CVI *p* = 0.013), meanwhile, no significant changes from basal to stimulation phase were observed for indices of sympathetic activity. All the parameters of heart rate variability and parasympathetic activity remained activated in the post-stimulus phase compared to the stimulus phase. In the post-stimulus phase, sympathetic activity resulted in a significant reduction (LFn *p* = 0.037).

**Conclusion:**

Music can influence the basal activity of the fetal autonomic nervous system, enhancing heart rate variability, without changing fetal heart rate mean value. Music is enabled to induce a relaxation state in a near-to-term fetus, mediated by parasympathetic activation and by a parallel sympathetic inhibition.

## Introduction

The autonomic nervous system (ANS) is a key homeostatic regulator both in prenatal and post-natal life (1). The main functions are regulated by the ANS: heart and flow rate, breathing rate, kidney, and gastroenteric function, and the secretion of the main endocrine glands (1, 2). These physiological functions depend on the degree of activation and maturation of the two main components of the ANS, namely the sympathetic and parasympathetic systems. The sympathetic system is responsible for the “fight-or-flight” response. Meanwhile, the parasympathetic nervous system is responsible for relaxation and moderating control over the sympathetic system, hypothalamic-pituitary-adrenal axis, and, when previous adaptive responses fail, it mediates the death-frightening behaviors as described by Porges in the “polyvagal theory” (2–4).

Fetal and neonatal wellbeing, as like fetal outcomes at birth, is strongly linked with ANS function. During fetal adaptation to postnatal life, ANS acts as the main regulator of the new-born cardiovascular and respiratory systems (2). At birth, both sympathetic activation and parasympathetic modulation play a fundamental role in increasing and stabilization of arterial blood pressure, heart rate, respiratory rate, and catecholamine level (2, 3, 5). Some of the most serious complications affecting new-born present an altered development of the ANS (6–8). When ANS activity is impaired, the risk of fetal death and sudden infant death outcomes increases (2, 8). In addition, children with such complications have a higher risk of developing chronic diseases (such as diabetes, hypertension, renal failure) (2, 9). Many studies underlined that ANS maturation, and, in particular, parasympathetic activation, seems to have a critical role in behavior, social communication, recognition, memory, mood, and adaptive responses to stress (2, 4, 10, 11). Then, we know that some autism spectrum disorders, rather than other severe diseases that afflict infants are often linked to impaired ANS development (12). This concept is of greater importance in preterm infants where altered ANS function may be compounded by immature development of the ANS itself and of the target organs (7).

For these reasons, it is important to identify markers and tools useful to evaluate and activate fetal ANS activity, to assess the neural maturity of a fetus, and to project clinical intervention about abnormal maturation of ANS management.

Several techniques have been studied to asset the quality of ANS activity in fetal life. Since the autonomic capacity is mirrored in cardiovascular regulation, a marker widely used for evaluating ANS activity is Heart Rate Variability (HRV) (1, 3, 9, 10, 13, 14). Analysis of HRV is used as a standard method for assessing ANS functions in adults. The association between HRV and autonomic function was first reported in the 1970s (15). HRV is an index of neurovisceral integration and organism self-regulation ability (16). Heart rate (HR) is mainly controlled by sinus node pacing. HRV represents the amount of HR fluctuation around the mean value of HR and is caused by continuous changes in the sympathetic/parasympathetic balance. In adulthood, HRV evaluation can be performed by statistical analysis (time-domain parameters), and spectral analysis (frequency-domain parameters) of R-R intervals obtained by ECG (17–20). Time-domain parameters quantify the amount of variability in R-R intervals, meanwhile, frequency-domain parameters estimate the distribution of absolute or relative power into the four specific frequency bands. HRV-related parameters can be extracted effectively from short-term electrocardiographic collection (5 min) (21, 22). N-N interval is another way of saying R-R interval, the letter N is used to distinguish that these statistics are intended to derive from “normal” R-R intervals, or R-R intervals which represent normal cardiac timing and are free from artifacts (23).

The evaluation of HRV is used as a predictor in a wide variety of disorders, like stroke, multiple sclerosis, end-stage renal disease, diabetes mellitus, ischemic heart disease, particularly myocardial infarction, cardiomyopathy, patients awaiting cardiac transplantation, valvular heart disease, and congestive heart failure (17–20).

Recently, some authors proposed fetal cardiomagnetography (fCMG) for the study of fetal ANS activity. The fMCG analysis allows obtaining time-domain and frequencies-domain parameters that reflect general maturation, adaptation, and organization of fetal ANS. Although, this method is quite expensive, not very usable in maternity care, and potentially dangerous if used routinely (1, 2, 24–26).

Our research group developed a non-invasive method of data collection of the fetal heart activity which could be considered equal to fCMG in providing useful data on ANS activity, called Fetal Autonomic Nervous sysTem Evaluation (FANTE) (27, 28). This technique consists of applying electrodes on the maternal abdomen to record joint maternal and fetal ECG signals. After data collection, fetal ECG signals are extracted and re-elaborated. This analysis allows the evaluation of time-domain and frequency-domain parameters related to ANS branches activity (27–29). With FANTE, we are able to assess the development of the ANS during fetal life, through the maternal abdomen, in an absolutely non-invasive, safe, and repeatable way. At the same time, it is possible to evaluate any changes in ANS activity after specific stimulations.

Acoustic stimulation and music have been used over the years in order to evaluate fetal and new-born well-being (30–34). Goodlin and Schimidt (35) first showed that fetuses with poor HR patterns that failed to respond to external sound were at increased risk for perinatal mortality or neurological impairment. Previous studies on anencephalic fetuses suggested that HR response to vibratory and acoustic stimulation is related to ANS activity (36, 37). Animal studies had revealed that a developed auditory apparatus is a necessary condition to obtain a fetal response (38–41). The human fetal auditory response begins about 26 weeks of gestation (30, 31, 34). Music played in the external environment is recognizable *in utero* (42). The ability of processing music involves a complex network of cortical and subcortical areas, and it has proved to be innate (34). Only around 32–33 weeks the maturation of the olivocochlear system allows relaying the information from the periphery to the cortex, for the elaboration of complex auditory stimuli such as music (43, 44).

Aim of this study was to determine the effect of music stimulation in normal fetuses from 32 to 38 weeks of gestation, through the analysis of temporal and frequency parameters related to ANS activity extracted from HR and HRV, recorded with the use of FANTE.

## Materials and Methods

### Selection Criteria

This study was carried out at the Gynecology and Obstetrics Department of the University of Pisa. From February to April 2021, we enrolled 32 pregnant women between the 32nd and 38th week of gestation. Women were enrolled during prenatal screening ultrasounds, and they carried a singleton and uncomplicated pregnancy. Exclusion criteria were multiple pregnancies, fetal malformations, drug abuse. Exit criteria were intrauterine fetal death, intrauterine restriction, and withdrawal of consent.

All participants received a FANTE data collection every week from the 32nd to the 38th week of gestation.

### Fetal Autonomic Nervous sysTem Evaluation Data Collection

**FIGURE 1:**
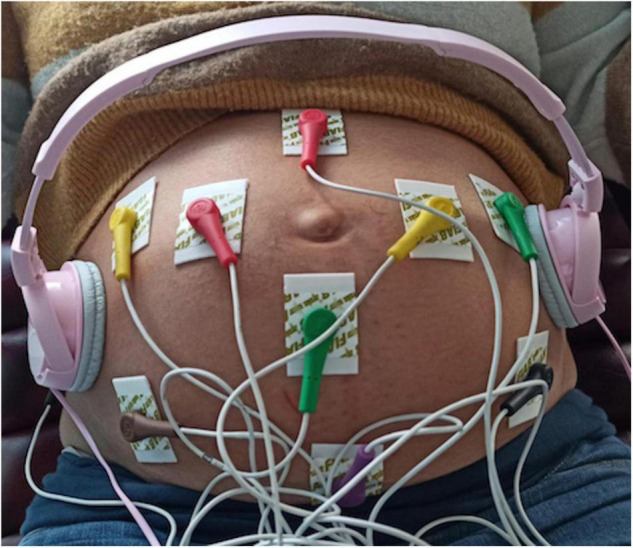
Scheme of 10 electrodes placed on the maternal abdomen during musical stimulation.

All FANTE data collections were acquired using a standard 10 derivation electrocardiograph (Cardioline S.p.A.) suitably modified. We used ten standard electrodes. The peripheral electrodes (Left, Right, and Foot) were placed on the maternal abdomen in a triangle around the navel (orange) with lower apex, while the precordial ones (C1, C2, C3, C4, C5, C6) were put on an external circle (Figure 1). The Neutral electrode was on the right side of the patient’s abdomen. The software was configured so that the system acquired and wrote on disk 8 signals (2 bipolar and 6 referenced to the centroid of the three L, R, and F leads) at 1,000 sps with a resolution of 0.763 uV. This high resolution is needed to resolve the very small voltage changes related to fetal cardiac activity.

### Music Stimulus

Consistently with previous studies, the effect of the auditory stimulus on fetuses and newborns was verified using classic music (44–46). For this reason, we choose as music stimulus “Clair de lune,” Debussy from London Symphony Orchestra.” The music stimulus was played through headphones (stereo Headphones MDR-ZX110, Sony Corporation, Tokyo, Japan) placed on the mother’s abdomen to avoid maternal acoustic interferences (Figure 1).

In our study, we decided to apply a music stimulus with a sound intensity under the level consider detrimental (85 dB) to avoid any possible harmful effect on the fetuses (47).

Moreover, in a previous study by Gagnon et al. (36), the authors attribute part of the elicitation effect of acoustic stimulation on HR to the contextual activation of cutaneous vibratory and tactile nervous pathways mediated by Meissner’s corpuscles (80 dB). To avoid this confounding factor and we used an average sound intensity of the music stimulus of 50.8 dB (range 39–84 dB).

### Experimental Design

To all patients, FANTE multiple data collections were proposed. The data collections were performed in the same room, in supine position, without other acoustic interference.

In every session, we obtain a basal FANTE data collection (5 min), a stimulus FANTE data collection (10 min), and a subsequent post-stimulus FANTE data collection (5 min).

### Fetal ECG Extraction and Analysis

Fetal ECG extraction and subsequent analysis from FANTE were achieved with a dedicated analysis software based on an algorithm developed and tested by the group of IFC-CNR (28, 48, 49). The algorithm automatically extracts fetal ECG, starting from maternal multichannel abdominal signals even in critical conditions (noise, movement of the mother). This algorithm consists of finding the linear combination of the acquired ECGs, which maximizes a quality index that summarizes the pseudo-periodicity and temporal characteristics of the fetal QRS complex. From the extracted fetal ECG, the RR series of the fetus was calculated as the difference among adjacent QRSs. In this study, we applied a procedure to remove heartbeats of non-sinus origin and artifacts in the RR series as previously described by Billeci et al. (23).

### Quality Assessment

From the initial sample of 32 mothers, 19 were excluded because their FANTE data collections were too noisy, and our method was unable to extract the fetal ECG and the obtained fetal RR series was meaningless. To exclude these cases from the analysis, we define a quality index. The Bad Index (BI) consider:

- the trimmed mean of the absolute successive differences of RR.
- the trimmed mean of the absolute successive second differences of RR.
- the ratio between the number of matched positions (of fetal QRS and maternal QRS) and the total number of QRS.
- a non-linear correction factor that takes accounts for the probability distribution of the mean fetal RR.

For this analysis, we consider FANTE data collection suitable for the analysis with a BI < 0.1 (threshold selected empirically). It is worth explaining that, at this gestational age, the execution of FANTE is achievable in 100% of cases. However, given the limited number of patients recruited, to obtain the best possible data quality, we included only FANTE data collection session in which BI < 0.1 in all three phases. This preliminary assessment led to the final sample of 13 mothers. Some of these mothers had multiple data collections in different gestational ages, leading to a sample of 20 signals.

The selected RR series was first screened for artifacts, uterine contractions, arrhythmias and then processed to compute temporal and frequency indices.

### Heart Rate and Heart Rate Variability Indices

The following indices were extracted from the RR series:

- Time-domain features of HRV:

• fHRm: fetal HR mean.
• RRsd (ms): standard deviation of RR intervals, index of total HRV;
• ANNsd (ms): standard deviation of the Average NN intervals for each 5 min segment of a 24 h HRV registration, index of total HRV;
• SD1 (ms): standard deviation perpendicular to the line of the identity of Poincairè Plot; short term variability, mainly controlled by parasympathetic tone;
• SD2 (ms): standard deviation along the line of the identity of Poincairè Plot; long term variability, index of total HRV;
• CVI (ms^2^): log10 (SD2xSD1); Cardiac Vagal Index, the contribution of the parasympathetic nervous system to cardiac regulation.

- Frequency-domain features of HRV:

• Ptot (ms^2^): total power of fetal RR series, index of total HRV, mainly controlled by sympathetic tone;
• LFn: spectral power in the low-frequency band (0.04–0.15 Hz) normalized for the total power; low-frequency band mainly controlled by sympathetic tone;
• HFn: spectral power in the low-frequency band (0.15–0.4 Hz) normalized for the total power; low-frequency band mainly controlled by parasympathetic tone.

### Statistical Analysis

GraphPad Prism 7 (GraphPad Software Inc., San Diego, CA, United States) program was used for data analysis and graphic presentations.

The parameters assessed in women have been included in the table. Category variables have been represented as frequencies and percentages, while continuous variables were represented as averages, standard deviations (SD), and interquartile ranges.

The normality of the variables was determined using the Shapiro–Wilk normality test. In accordance, ordinary or repeated measures (RM) one-way ANOVA, followed by paired sample Tukey’s multiple comparisons test, was performed when comparing features extracted from fetal RR series in every session for each patient.

For all comparisons, the values of *p* < 0.05 were considered statistically significant.

### Ethical Approval

This study was carried out in agreement with the recommendations of the Good Clinical Practice (ICH/GCP), Ministerial Decree of 1997. The protocol was approved by the Regional Ethics Committee for Clinical Trials, Tuscan North West Wide Area. All subjects gave written informed consent by the Declaration of Helsinki (clinical trial number 31927).

## Results

### Population

The demographic and clinical characteristics of the population are summarized in Table 1.

**TABLE 1:** Population features analysis and description (*n* = 13).

Population (n)	13
Age (mean ± SD)	34.2 ± 4.5
BMI kg/m2 (mean ± SD)	25.2 ± 5.0
Gestational diabetes (n,%) Diet therapy	1 (7.7%) 1 (7.7%)
Delivery mode (n,%) Vaginal delivery Elective C-section Unknown	9 (69.2%) 2 (15.3%) 2 (15.3%)
Apgar 5 min (n,%) 10 9 Unknown	2 (15.3%) 10 (76.9%) 1 (7.7%)

*SD, standard deviation.*

All women had a full-term delivery. No newborn needed neonatal resuscitation.

### Music Does Not Change the Mean Value of Fetal Heart Rate but Increases Fetal Heart Rate Variability

**FIGURE 2:**
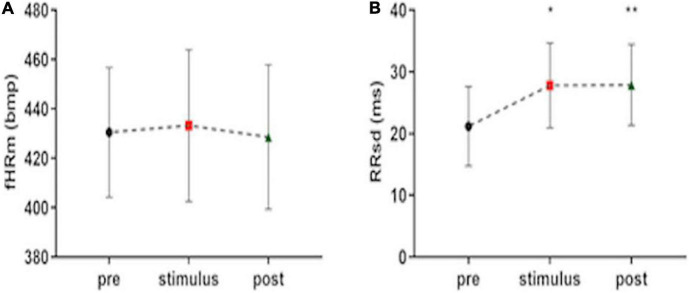
Fetal heart rate mean **(A)** and heart rate variability, expressed ad RRsd **(B)** during FANTE data collection. pre, basal FANTE data collection; stimulus, stimulus FANTE data collection; post, post-stimulus FANTE data collection. *significant at the alpha level *p* < 0.05; **significant at the alpha level *p* < 0.01.

Music presents different effects on fetuses. The value of fHRm does not change during the stimulus phase and in the post-stimulus. However, fetal HRV statistically increases during the stimulus phase and this modification persists in the post-stimulus FANTE data collection (Figures 2A,B; Table 2).

**TABLE 2:** Features extracted from the RR series in the different phases of the protocol.

	Pre Mean ± SD	Stimulus Mean ± SD	Post Mean ± SD	Pre vs. Stimulus	Pre vs. Post	Stimulus vs. Post
fHRm (bpm)	430.5 ± 26.23	433.3 ± 30.78	428.6 ± 29.25	*p* = 0.826	*p* = 0.923	*p* = 0.692
RRsd (ms)	21.18 ± 6.43	27.82 ± 6.88	27.89 ± 6.55	*p* = 0.037*	*p* = 0.009**	*p* = 0.99
ANNsd (ms)	16.41 ± 5.32	22.93 ± 7.86	21.73 ± 7.37	*p* = 0.036*	*p* = 0.015*	*p* = 0.832
Ptot (ms2)	441.1 ± 254.2	571.7 ± 255.9	680.6 ± 290.8	*p* = 0.207	*p* = 0.019*	*p* = 0.346
SD2 (ms)	27.53 ± 8.09	36.57 ± 10.19	36.40 ± 9.59	*p* = 0.039*	*p* = 0.009**	*p* = 0.997
CVI (ms2)	2.34 ± 0.35	2.65 ± 0.19	2.59 ± 0.30	*p* = 0.013*	*p* = 0.033*	*p* = 0.695
SD1 (ms)	477.5 ± 325.3	548.3 ± 292.2	691.6 ± 329.5	*p* = 0.768	*p* = 0.051	*p* = 0.302
HFn (n.u.)	0.21 ± 0.22	0.21 ± 0.17	0.26 ± 0.25	*p* = 0.964	*p* = 0.569	*p* = 0.498
LFn (n.u.)	0.29 ± 0.13	0.27 ± 0.11	0.19 ± 0.09	*p* = 0.88	*p* = 0.012*	*p* = 0.037*

SD, standard deviation; ms, milliseconds; ms2, millisecond squared; n.u., normalized unit; pre, basal FANTE data collection; stimulus, stimulus FANTE data collection; post, post-stimulus FANTE data collection. *significant at the alpha level p < 0.05; **significant at the alpha level p < 0.01.

### Heart Rate Variability Change Depends Mainly on the Activation of Parasympathetic Activity

**FIGURE 3:**
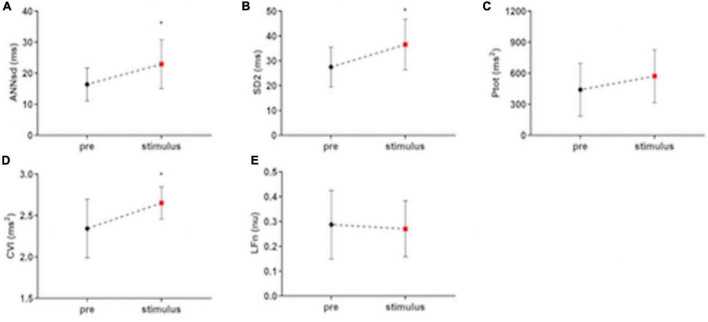
The impact of musical stimulation on index-linked to ANS activity. **(A)** ANNsd (ms): standard deviation of the Average NN; **(B)** SD (ms): standard deviation perpendicular to the line of the identity of Poincairè Plot; short term variability, mainly controlled by parasympathetic tone; **(C)** Ptot (ms2): total power of fetal RR series, index of total HRV, mainly controlled by sympathetic tone; **(D)** CVI (ms2): Cardiac Vagal Index, the contribution of the parasympathetic nervous system to cardiac regulation; **(E)** LFn: low-frequency band (0.04–0.15 Hz) normalized for the total power; low-frequency band mainly controlled by sympathetic tone; pre: basal FANTE data collection; stimulus: stimulus FANTE data collection; post; post-stimulus FANTE data collection. *significant at the alpha level *p* < 0.05; **significant at the alpha level *p* < 0.01.

All the indices of the fetal heart variability significantly increase during the stimulus phase data collection compared to basal phase: RRsd (*F* = 6.71, *p* = 0.037, *R*^2^ = 0.26) (Figure 2B), ANNsd (*F* = 5.55, *p* = 0.036, *R*^2^ = 0.23), SD2 (*F* = 6.40, *p* = 0.039, *R*^2^ = 0.25), Ptot (*F* = 4.91, *p* = 0.019, *R*^2^ = 0.25) (Figures 3A–C). CVI, marker of parasympathetic activation, also statistically increases (*F* = 0.94, *p* = 0.013, *R*^2^ = 0.29) (Figure 3D) while other indices of parasympathetic activity (SD1 and HFn) do not change (Table 2).

LFn, the low-frequency band mainly controlled by sympathetic tone, does not modify in the stimulus phases (*F* = 5.16, *p* = 0.88, *R*^2^ = 0.21) (Figure 3E).

### Music Ending Does Not Change Heart Rate Variability Compared to Stimulation

**FIGURE 4:**
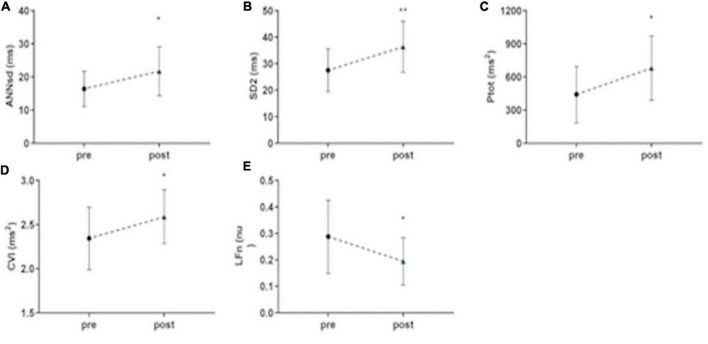
Persistent short-term musical modulation of ANS activity. **(A)** ANNsd (ms): standard deviation of the Average NN; **(B)** SD (ms): standard deviation perpendicular to the line of the identity of Poincairè Plot; short term variability, mainly controlled by parasympathetic tone; **(C)** Ptot (ms2): total power of fetal RR series, index of total HRV, mainly controlled by sympathetic tone; **(D)** CVI (ms2): Cardiac Vagal Index, the contribution of the parasympathetic nervous system to cardiac regulation; **(E)** LFn: low-frequency band (0.04–0.15 Hz) normalized for the total power; low-frequency band mainly controlled by sympathetic tone; pre: basal FANTE data collection; stimulus: stimulus FANTE data collection; post; post-stimulus FANTE data collection. *significant at the alpha level *p* < 0.05; **significant at the alpha level *p* < 0.01.

All the parameters, which express ANS modulation of HRV, remain unchanged during the post-stimulus phase compared to stimulation phase: RRsd (*p* = 0.99) (Figure 2B), ANNsd (*p* = 0.832), SD2 (*p* = 0.997), Ptot (*p* = 0.346) (Figures 4A–C), but LFn. Instead, LFn (which expresses mainly the sympathetic tone) results in a significant reduction in the post-stimulus phase, in comparison to the stimulus phase (*p* = 0.037) (Figure 4E). CVI, controlled by parasympathetic tone, keeps being activated in the post-stimulus phase (*p* = 0.695) (Table 2).

### Music Stimulus Induces Heart Rate Variability Modification Even When the Music Stops

Considering ANS signaling before and after music stimulation, we can note an important role of music as a stimulus of ANS activation. When the music stops, ANS modification persists in the following 5 min. Comparing ANS activity in the basal phase and the post-stimulus phase, the parameters showed a significant increase of the HRV (RRsd, ANNsd, Ptot) (Figures 2B, 4A–C) modulated by the increasing of parasympathetic activity (CVI) (Figure 4D) and by the parallel reduction of sympathetic tone (LFn) (Figure 4E; Table 2).

## Discussion

In this paper, we presented the effects of a short musical stimulus (10 min) on the activity of the fetal ANS, through the modulation of sympathetic and parasympathetic signaling. We registered this modulation through external sensors placed on the maternal abdomen as already shown in a previous work (24).

Music induces a modification of the parameters which regulate HRV (RRsd, ANNsd, Ptot), without a significant change in fetal heart rate.

This modulation persists as a short-term effect even after the music stops, for the entire length of the post-stimulus FANTE data collection (about 5 min). In our opinion, this demonstrates that music, at the intensity we used (mean 50.8 dB), determinates the activation of ANS without inducing a fight-or-flight reaction, sympathetically mediated, as a simple sound stimulus does. The music-dependent relaxation-state of the fetus is achieved by the increase of CVI through the activation parasympathetic system (2, 3).

It has been widely demonstrated that acoustic stimulation can influence HR activity (30–32, 36). Various external stimuli, and in particular vibratory and acoustic stimulation have been used over the years to evaluate fetal well-being (30–34). In the study conducted by Kisilevsky et al. (44), fetuses exposed to vibroacoustic stimulation at least 95 dB, showed a gradual heart rate acceleration. The same effect was observed by Al-Qahtani et al. (50) on fetuses between 37 and 40 weeks exposed to human voices higher than 105 dB. Those data were confident with the ones by Lecanuet et al. (51), in which term-fetuses responded to acoustic stimulus by increasing the ratios and amplitudes of accelerations and reducing deceleration ratios. Notably, the authors highlight that acoustic stimulus with lower sound intensity could elicit cardiac decelerations in near-to-term fetuses. This bimodal effect of acoustic stimulation has been considered by the authors as an expression of the defense and orientating response described in Sokolov’s model of information processing influenced by autonomic feedback.

Defense response is typically elicited by high-intensity stimulus and it determinates a sympathetic activation, meanwhile orienting response is activated by low-intensity stimulus and it reflexes a parasympathetic activation. Based on Sokolov’s model, those two responses were related to cardiac change (52).

In our study, the application of a music stimulus did not change the value of fetal heart rate. In accord with our results, D’Elia et al. (30) did not observe a change in fetal heart rate between 36 and 42 weeks after a vibroacoustic stimulation using an artificial larynx with an averaged sound intensity level of 68 dB. However, the low intensity of the music we used is not irrelevant. This stimulus is decoded by the fetal ANS and it determines a re-modulation of parasympathetic tone, in terms of increase, and of sympathetic tone, in terms of decrease. In particular, other studies using classic music showed a distressed effect of this type of stimuli on the ANS in the fetus, by using cardiotocography (45), or in newborns (46, 53). Therefore, this type of music seems to have a relaxing role for the fetal ANS, mediated both by a parasympathetic and sympathetic system, without determining a modification of the fHRm.

In the study conducted by Lecanuet et al. (54), the author observed that the magnitude of fetal cardiac and motor response to an acoustic stimulus was related to the basal HRV pattern. In our data, we did not observe any relation between the basal value of HRV indices (RRsd, ANNsd, SD2, Ptot), parasympathetic index (CVI), and their increase during the stimulus phase. We can only hypothesize that the effect of music on fetal ANS is not influenced by fetal behavioral state and does not depend on ANS basal tone.

From a translational point of view, the demonstration of the efficacy of an external stimulus in modulating the ANS signal is innovative and very promising, especially in those cases of incomplete development of the ANS.

For the first time, we demonstrated that, with music, it is possible to modulate fetal ANS activity in the short term, opening potentially significant scenarios from a therapeutic point of view. For example, in pregnant women with threatened preterm labor (where it is expected the birth of infants with delayed development of the ANS) rather than in term fetuses with potential risks of associated diseases (fetal growth restriction, preeclampsia, etc.), the use of music in the prenatal period, through *ad hoc* stimulation, could help the activation of the ANS and therefore its development.

On the other hand, we demonstrated music determines an early modulation of the whole autonomic activity, although, with our data, it is still not possible to verify if these modulating effects determine a long-term modification of fetal ANS basal activity.

The missing data about the possible long-term effect of music on the fetus is the real point of the weakness of our study. We were not able to demonstrate if this stimulus could have a real and positive effect on fetal ANS. This needs further investigation to clarify if we can long-term modulate fetal development and wellness with acoustic stimulus, perhaps repeated several times, at different time points of the pregnancy.

Another limitation of the study is the small sample size. In our previous article, we have been able to retrieve fetal ECG signals in the 62.5% of data collections. In the present study, the efficiency of the FANTE data collection was a little bit lower, since from 32 weeks of gestation to term, the presence of vernix caseosa can limit the possibility of fetal ECG signal extraction (27). Moreover, our decision to consider only FANTE data collection sessions in which BI < 0.1 in all three phases to obtain high-quality data further reduced the sample size. Finally, as described, our study aimed to verify the effect of music on near to term fetuses, hypothetically healthy. This was decided to avoid possible interfering factors related to fetal or maternal diseases which could alter ANS response in some way. Therefore our data are strictly linked to the wellness of the fetus. We have no data on “complicated” pregnancies and it is not possible to hypothesize beneficial effects in this type of patient.

This study concludes that music can modulate fetal ANS basal tone in the short term, enhancing HRV, without changing fHRm. In particular, music enables to induce a relaxation state in a near-to-term fetus, mostly mediated by parasympathetic activation.

## Data Availability Statement

The raw data supporting the conclusions of this article will be made available by the authors, without undue reservation.

## Ethics Statement

The studies involving human participants were reviewed and approved by the Regional Ethics Committee for Clinical Trials, Tuscan North West Wide Area. The patients/participants provided their written informed consent to participate in this study.

## Author Contributions

PM, LB, and MV conceived and designed the study and coordinated the project. FM, AC, and GI performed the study and collected the data. LB and MV were responsible for the formal analysis. FM and MM-G were responsible for the data curation and provided the support in data analysis and visualization of results. PM, LB, and FM were involved in the interpretation of the data. FM prepared the first draft of the manuscript. PM, LB, MV, and TS revised it critically. All authors have given their approval for this version to be published.

## Conflict of Interest

The authors declare that the research was conducted in the absence of any commercial or financial relationships that could be construed as a potential conflict of interest.

## Publisher’s Note

All claims expressed in this article are solely those of the authors and do not necessarily represent those of their affiliated organizations, or those of the publisher, the editors and the reviewers. Any product that may be evaluated in this article, or claim that may be made by its manufacturer, is not guaranteed or endorsed by the publisher.
